# Case Report: Compound Heterozygous Variants of the *MAN1B1* Gene in a Russian Patient with Rafiq Syndrome

**DOI:** 10.3390/ijms231810606

**Published:** 2022-09-13

**Authors:** Irina Zh. Zhalsanova, Ekatherina G. Ravzhaeva, Anna E. Postrigan, Gulnara N. Seitova, Daria I. Zhigalina, Vasilisa Yu. Udalova, Maryana M. Danina, Ilya V. Kanivets, Nikolay A. Skryabin

**Affiliations:** 1 Tomsk National Research Medical Center, Research Institute of Medical Genetics, 634050 Tomsk, Russia; 2 Genomed Ltd., 115093 Moscow, Russia

**Keywords:** Rafiq syndrome, congenital disorders of glycosylation, *MAN1B*, next-generation sequencing

## Abstract

Rafiq syndrome (RAFQS) is a congenital disorder of glycosylation (CDG) that is caused by mutations in the *MAN1B1* gene and characterized by impaired protein and lipid glycosylation. RAFQS is characterized by a delay in intellectual and motor development, facial and other dysmorphism, truncal obesity, behavior problems, and hypotonia. We describe a Russian patient with delayed intellectual and motor development, a lack of speech, disorientation in space and time, impaired attention and memory, and episodes of aggression. Screening for lysosomal, amino acid, organic acid, and mitochondrial disorders was normal. The patient was referred for the targeted sequencing of the “Hereditary Metabolic Disorders” panel. The genetic testing revealed two heterozygous pathogenic variants in the *MAN1B1* gene: the previously reported c.1000C > T (p.Arg334Cys) and the novel c.1065 + 1 G > C. Thus, the patient’s clinical picture and genetic analysis confirmed RAFQS in the patient.

## 1. Introduction

Rafiq syndrome (RAFQS) (OMIM #614202) is a rare autosomal recessive disorder that belongs to the type II congenital glycosylation disorders (MAN1B1-CDG). The molecular cause of the syndrome is pathogenic variants in the *MAN1B1* gene (mannosidase alpha class 1B member 1) on chromosome 9q34.3. The gene encodes an enzyme that belongs to glycosyl hydrolase family 47 and that is localized to the Golgi apparatus—the endoplasmic reticulum mannosyl-oligosaccharide alpha-1,2-mannnosidase—and it is involved in N-glycosylation in the secretory pathway [1]. A defect in the *MAN1B1* gene affects the N-glycosylation of the protein and disrupts the Golgi’s overall morphology. Mutations lead to desynchronization and the cleavage of misfolded glycoproteins through protein degradation associated with the endoplasmic reticulum [2].

Mental retardation, motor-developmental delay, a characteristic facial phenotype, truncal obesity, and hypotonia characterize RAFQS. In most patients, prominent eyebrows with lateral thinning, downward-sloping palpebral fissures, a bulbous nose tip, large ears, and a thin upper lip represent the facial phenotype [3]. Establishing a diagnosis is often difficult because of the low prevalence of the disease, the heterogeneity of the clinical spectrum, and the absence of pathognomonic clinical symptoms. To date, researchers have described over 40 patients of MAN1B1-CDG worldwide, and about 230 variants of the gene nucleotide sequence are known, of which about 20 are pathogenic/likely pathogenic. However, this syndrome has not been diagnosed in Russia. [4].

Here, we report the first Russian patient with Rafiq syndrome.

## 2. Patient Report

A 2.4-year-old Russian boy was referred because of psychomotor and speech disability. The main symptoms were complaints of poor speech development, a poor understanding of addressed speech, and a lack of skills due to age. He was born from the fourth pregnancy, third childbirth (1 abortion). The proband has a healthy brother and sister. A sibling’s blood sample was not available. The pregnancy proceeded against the background of maternal subclinical hypothyroidism. Emergency childbirth took place at 37 weeks of pregnancy. Weight at birth was 2910 g and length 49 cm. The Apgar score was 7/8. Further development was characterized by a delay in early motor development. From the age of 4 months, he could hold his head in the prone position. At 1 year he could sit without help and at 1.3 years walk with support. Biochemical studies showed normal results except for hypoglycemia. Serum transferrin isoelectrofocusing was not performed. The leucocyte karyotype was normal as well as methylation-specific PCR of the *FMR1* gene promotor. During the functional tests, no focal or generalized pathological activity was detected, and no substantial interhemispheric asymmetry. No specific forms of activity were registered. A brain MRI revealed high signal diffuse areas in the subcortical regions against the background of incomplete myelination. Upon the first physical examination in our inpatient department at 2.4 years old, the patient had a height of 89 cm (50th centile) and a weight of 12 kg (50th centile). He could not speak and had difficulty in understanding speech. He could walk and run without support. He showed aggressive behavior. Facial dysmorphism included a flat wide nasal bridge. Fat distribution and respiratory, cardiovascular, digestive, and urogenital systems were normal.

## 3. Results

### 3.1. Next-Generation Sequencing

The patient underwent targeted sequencing of the “Hereditary Metabolic Disorders” panel, 561 genes associated with metabolic disorders. The genetic testing identified two heterozygous variants in the *MAN1B1* gene: c.1000C > T and c.1065 + 1 G > C.

Missense variant c.1000C > T was revealed in exon 7 of the *MAN1B1* gene (chr9:137101088C > T), which has previously been described as pathogenic. The variant results in the replacement of arginine by cysteine at position 334 (p.Arg334Cys; NM_016219.5). The missense variant c.1000C > T has been described as pathogenic [1,3,4,5,6]. Thus, we can regard this nucleotide sequence variant as pathogenic.

The other variant, c.1065 + 1 G > C, is in the canonical splicing site of exon 7 of the *MAN1B1* gene (chr9:137101154G > C). This mutation leads to the disruption of RNA splicing and the formation of a defective protein product, or its complete absence. Loss-of-function in the *MAN1B1* gene is a known mechanism of disease (the gene has 25 pathogenic LOF variants and gnomAD Loss-of-Function Observed/Expected = 0.56 is less than 0.755), associated with Autosomal Recessive Intellectual Developmental Disorder, Autosomal Recessive Non-Syndromic Intellectual Disability, and Rafiq Syndrome. The described nucleotide sequence variant was not registered in the control samples of the Genome Aggregation Database, Exome Aggregation Consortium, or 1000 Genomes Project. The position of this variant is strongly conserved (phyloP100 way = 7.73 is greater than 7.2) and this variant is predicted to be spliced (scSNV ADA Boost score = 1 is greater than 0.708). The variant is described in the VarSome database. The pathogenic computational verdict was based on seven pathogenic predictions from BayesDel noAF, BayesDel_addAF, CADD, EIGEN, EIGEN PC, FATHMM-MKL, MutationTaster, vs. no benign predictions. Thus, we should regard this variant as pathogenic.

### 3.2. Sanger Sequencing

**Figure 1:**
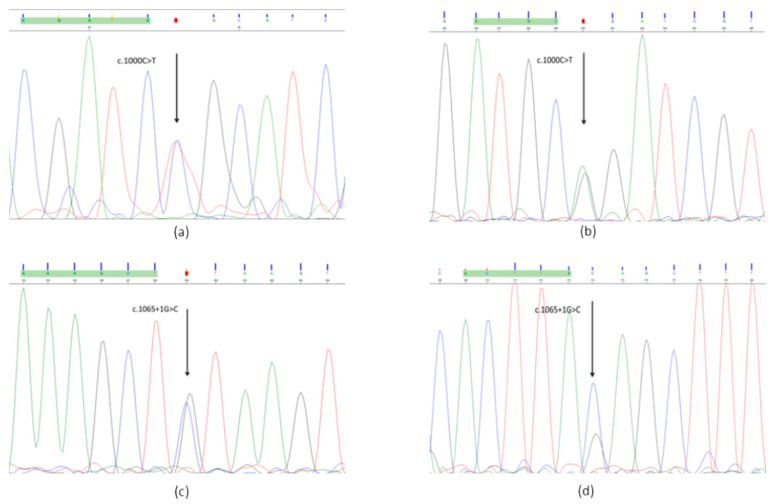
Sanger sequencing: (**a**) c.1000C > T forward primer; (**b**) c.1000C > T reverse primer; (**c**) c.1065 + 1 G > C forward primer; (**d**) c.1065 + 1 G > C reverse primer.

According to the Sanger sequencing, the patient had both mutations (Figure 1*)*. The c.1000C > T variant was present in the mother. A father’s blood sample was not available.

## 4. Discussion

In this study, we describe a Russian patient with two variants in the *MAN1B1* gene. This may be the first reported patient with Rafiq syndrome in the Russian Federation to date. The patient had moderate mental and motor disability, and aggressive behavior. He had no other symptoms of MAN1B1-CDG patients such as truncal obesity, dolichocephaly, and joint hypermobility.

Researchers have repeatedly described the identified mutation (c.1000C > T, p.R334C) in patients with mental retardation. Table 1 summarizes the patient’s clinical features currently registered with the c.1000C > T mutation associated with the Rafiq syndrome. The first mention of this mutation was in a 2011 Rafiq study on an Iranian family [1]. Other cases of homozygous carriage of the c.1000C > T mutation include three patients of Turkish origin. The first case was an 11-year-old girl; besides the main clinical manifestations described in Table 1, the patient has joint hypermobility and skin laxity. The second and third cases included siblings (aged 24 and 18 years) whose parents were consanguineous [4]. Hoffjan also provides information on a consanguineous Turkish family with a homozygous c.1000C > T mutation [5]. Van Scherpenzeel described a 15-year-old patient with the indicated features [6]. Researchers identified two heterozygous mutations in the *MAN1B1* gene, c.1000C > T, p.Arg334Cys, and c.761_764del, p.Ile254Thrfs*20, in a 10-year-old girl of European origin. The patient had a slight developmental delay, marked outbreaks of aggressive behavior, open palpebral fissures, and hypertelorism representative of facial dysmorphism. Hyperphagia developed with age. The patient’s intellectual development was preserved [3]. Kasapkara, in 2021, described a 3-year-old patient from a Turkish family with developmental delay and hypotension with homozygous variant c.1000C > T, which was inherited from the parents [7].

The second mutation, c.1065 + 1G > C, has not been previously described in publications and is not registered in databases; however, according to the clinical guidelines for the interpretation of NGS data, we can consider this variant to be a new pathogenic variant that is responsible for the development of RAFQS clinical symptoms.

## 5. Materials and Methods

### Next-Generation Sequencing and Sanger Sequencing

The analysis was performed with next-generation sequencing using paired-end reads. For the sample preparation, the technique of the selective capture of DNA regions belonging to the coding regions of the genes included in the panel (561 genes associated with hereditary metabolic diseases) was used. The sequencing data were analyzed using an automated pipeline consisting of a read alignment to the reference human genome sequence (hg38), alignment postprocessing, variant calling, quality filtering, and the annotation of the identified variants with the canonical transcript of each gene. The pathogenicity of the variants was determined considering the ACMG recommendations.

The pipeline also includes the algorithm for the CNV (copy number variation) calling based on the read depth. The read length was 2 × 151 bp. The average coverage was 73×. The pipeline employs BWA MEM for sequence alignment, Freebayes for variant calling, SnpEff and SnpSift tools for variant annotation, using the default settings except for Freebayes where long haplotype calling was turned off. Raw calls were filtered based on sequencing depth (10 or above to pass filter) and strand bias (required presence of at least one alternative allele read on both DNA strands in calls where reference reads are present on both strands). The clinical relevance of selected variants was determined using OMIM, specialized disease databases (when available), and literature data.

Sanger sequencing around the identified variants was performed in both directions in the patient and his mother. The following primers for the PCR were selected: forward: CGTGGAGCCATCCATTTGT; reverse: CAAGGAAACGGGCATCACTC.

## 6. Conclusions

In conclusion, we described the first Russian patient with Rafiq syndrome including a novel variant. Thus, we expanded the clinical and molecular disease spectrum, and we presented the features of the clinical manifestation in the patient with a novel variant.

## Figures and Tables

**Table 1:** Patients’ clinical descriptions with a c.1000C > T mutation in other publications.

	Developmental and Intellectual Disability	Truncal Obesity	Behavioral Concerns	Seizures	Facial Dysmorphism	Genotype	Total (of Reported Features)
Rafiq, M.A. 2011 [1]	3/3	0/3	2/3	1/3	3/3	c.1000C > T/c.1000C > T	9/15 (60%)
Rymen, D. 2013 [4]	3/3	3/3	0/3	0/3	3/3	c.1000C > T/c.1000C > T	9/15 (60%)
Van Scherpenzeel, M. 2014 [6]	1/1	1/1	0/1	0/1	1/1	c.1000C > T/c.1000C > T	3/5(60%)
Hoffjan, S. 2015 [5]	3/3	3/3	0/3	0/3	3/3	c.1000C > T/c.1000C > T	9/15 (60%)
Balasubramanian, M. 2019 [3]	1/1	1/1	1/1	0/1	1/1	c.1000C > T/c.761_764del	4/5 (80%)
Kasapkara, C.S. 2021 [7]	1/1	1/1	0/1	0/1	1/1	c.1000C > T/c.1000C > T	3/5 (60%)
Current case	1/1	0/1	1/1	0/1	1/1	c.1000C > T/c.1065 + 1G > C	3/5 (60%)

## Data Availability

Due to the privacy policy, data are available upon request after approval from the patients.
